# Magnitude of risk factors for chronic noncommunicable diseases in adolescents and young adults in Brazil: A population-based study

**DOI:** 10.1371/journal.pone.0292612

**Published:** 2023-10-19

**Authors:** Charlise Fortunato Pedroso, Cristina Camargo Pereira, Agueda Maria Ruiz Zimmer Cavalcante, Rafael Alves Guimarães

**Affiliations:** 1 Federal Institute of Education, Science and Technology of Goiás, Goiânia Oeste Campus, Goiânia, Goiás, Brazil; 2 Institute of Tropical Pathology and Public Health, Federal University of Goiás, Goiânia, Goiás, Brazil; 3 Faculty of Nursing, Federal University of Goiás, Goiânia, Goiás, Brazil; ICMR-National Institute of Medical Statistics, INDIA

## Abstract

**Aim of the study:**

Estimate the magnitude and factors associated with risk factors for chronic noncommunicable diseases in adolescents and young adults in Brazil.

**Methods:**

Cross-sectional study that analyzed data from the 2019 National Health Survey. The population of interest was adolescents and young adults aged 15 to 24 years. Data were collected through individual interviews during home visits. Dependent variables included major risk factors for chronic noncommunicable diseases. Demographic and socioeconomic characteristics were used as independent variables. Multiple Poisson regression models were used to assess the relationship between independent variables and risk factors.

**Results:**

A total of 10,460 individuals (5,001 men and 5,459 women) were included. Regardless of sex, the most prevalent risk factors were insufficient fruit and vegetable consumption (92.6%) and leisure-time physical inactivity (43.3%). The prevalence rates of tobacco smokers, alcohol consumption once a month or more, and alcohol abuse were 8.9%, 28.7%, and 18.5%, respectively. Regular consumption of soft drinks and/or artificial juices was described by 17.2%. The prevalence of overweight was 32.5%. Young adults, males, and individuals with lower educational levels, of black race/skin color, with lower household income, and residents of urban areas had a higher prevalence for most risk factors. Differences in the determinants were found for some factors. Inequalities between Brazilian regions were recorded for seven of the nine factors analyzed. The most socioeconomically developed regions had the highest prevalence of most risk factors. The high magnitude of risk factors indicates a potential increase in the burden of chronic noncommunicable diseases in a future scenario for Brazil.

## Introduction

Chronic noncommunicable diseases (NCDs), including cardiovascular diseases, cancers, diabetes, and chronic respiratory diseases, are responsible for 74% of global mortality, causing 41 million deaths annually. NCDs are associated with high rates of premature morbidity and mortality, reduced quality of life, loss of productivity, and high economic costs for countries [1]. The prevalence and impact of these chronic conditions are more significant in low- and middle-income countries compared to high-income countries [2].

Brazil, the largest country in Latin America, has also shown high mortality rates from NCDs. In 2019, 1 million and 26 thousand deaths were due to NCDs, representing 75.0% of deaths. Most deaths from NCDs in the country occur prematurely in individuals aged 30 to 69 years [2].

The burden of NCDs is increasing in adolescents and young adults in developed and developing countries [3, 4]. Estimates from the Global Burden of Disease Study (GBD) 2019 of European Union member states revealed that NCDs accounted for 38.8% of all deaths in individuals aged 10 to 24 years [3]. Data show that 70% of potentially preventable deaths from NCDs in adults are the result of health-related behaviors initiated in childhood and adolescence [4]. Despite the multicausal nature involved in the etiology of NCDs, the main determinants of these diseases are behavioral risk factors, represented by tobacco use, alcohol use, unhealthy diet, physical inactivity, and overweight, which can lead to raised blood pressure, high serum cholesterol and blood glucose levels [2]. These factors are highly prevalent in adolescents and young adults and, when they coexist, increase the likelihood of developing NCDs [3, 4].

Existing literature has shown a high prevalence of risk factors for NCDs in adolescents and young people. A global study conducted with 587,565 adolescents aged 11–17 years found that the median prevalence by individual risk factor was highest for insufficient vegetable consumption (86.0%), insufficient fruit consumption (85.0%), and physical inactivity (85.0%). The median prevalence of tobacco use and alcohol use was 9.0% and 21.0%, respectively [5]. A survey carried out with Canadian students in grades 9 to 12 showed prevalence of current smokers and current binge drinkers of 8.8% and 25.5%, respectively. Moreover, the prevalence estimates of inadequate fruit and vegetable consumption, physical inactivity, and overweight were 93.6%, 89.4%, and 22.5%, respectively [6]. Research conducted with adolescents in four Caribbean countries (Dominican Republic, Suriname, Jamaica and Trinidad, and Tobago) also identified a high prevalence of behavioral risk factors for NCDs in participants aged 16 years and older, including tobacco use (14.2%), alcoholic beverage consumption (47.8%), inadequate fruit and vegetable consumption (84.0%), low physical activity (86.2%), and overweight (23.0%) [7].

In Brazil, few studies have investigated the prevalence of risk factors for NCDs in adolescents and young adults. The largest national survey, the Brazilian National Survey of School Health (PeNSE), was carried out in 2019 by the Ministry of Health among 125,123 school adolescents aged 13 to 17 years and estimated the following prevalence of risk factors: current smokers (6.8%), alcohol consumption ever in life (63.3%), alcohol abuse (6.9%), regular consumption of soft drinks (17.2%), ultra-processed food consumption (UPF) on the previous day to the survey (97.3%) and physical inactivity (8.7%) [8]. There is a lack of studies in Brazil investigating the determinants of risk factors for NCDs in adolescents and young people.

Importantly, addressing these conditions and risk factors is a key priority for achieving comprehensive health care for adolescents and young adults [3, 4]. The measurement and monitoring of these factors can contribute to implementing effective policies and agendas (such as the Strategic Action Plan for Tackle Chronic Diseases and Noncommunicable Diseases in Brazil 2021–2030 [9] and the 2030 Agenda for Sustainable Development [10]) and preventive strategies to reduce their prevalence in this population and, consequently, the prevalence of NCDs. However, in Brazil, few population-based studies have investigated the magnitude of risk factors in adolescents and young people. Previous research focused predominantly on adults, not presenting specific data for adolescents and young adults, especially information disaggregated by sex and for several factors (for example: UPF consumption and regular consumption of soft drinks and/or artificial juice). Additionally, there is scarce literature exploring associated factors of risk factors for NCDs in these populations. Thus, this study aimed to estimate the magnitude and factors associated with risk factors for NCDs in adolescents and young adults in Brazil.

## Materials and methods

### Data source

The present study used data from the National Health Survey (NHS) conducted in 2019 and 2020 in Brazil. The NHS is a cross-sectional, population-based household survey carried out by the Brazilian Institute of Geography and Statistics in partnership with the Ministry of Health [11].

Brazil is a country in South America with 5,570 municipalities divided into 26 states and the Federal District, where the national capital Brasília is situated. The federative units and the Federal District are grouped into five major regions: South, Southeast, Midwest, Northeast, and North. In 2019, the Brazilian population reached 210,147,125 inhabitants, with a Human Development Index of 0.765, Gross Domestic Product (GDP) per capita of BRL 35,161.70, and household income per capita of BRL 1,439 [12].

The primary purpose of the NHS was to provide information on the health determinants, conditions, and needs, contributing to the elaboration of public policies and greater effectiveness in health interventions in Brazil. Data collection occurred between August 2019 and March 2020 [11].

### Participants

The target population of the NHS consisted of representative samples of individuals aged 15 years or older residing in permanent private households in one of the federative units. Permanent private households are those built for the sole purpose of housing. Therefore, sectors with special or sparse populations were excluded from the sampling process, such as indigenous clusters, barracks and military bases, lodgings, campsites, prisons, long-stay institutions for the elderly, comprehensive care facilities for children and adolescents, convents, hospitals, settlement project farming communities and quilombola clusters [11]. In this study, the target population consisted of adolescents and young adults aged 15 to 24 years, living in permanent private households in Brazil.

### Sampling plan and sample

The sampling strategy of the NHS consisted of a three-stage conglomerate plan, with stratification of the primary sampling units (PSU). The PSU included the census tracts or sets of tracts, defined as the territorial unit, formed by a continuous area, located in a single urban or rural setting, with a defined dimension and number of households. The second stage consisted of selecting households in each PSU, constituting the secondary sampling units. The third stage corresponded to the selection of a resident aged ≥15 years, from each household, obtained from the list of residents during the interview. The selected resident included the Tertiary Sampling Units. The three stages were conducted through simple random sampling [11].

The sample size defined for the NHS considered the desired accuracy for estimating the main risk and protective factors for NCDs, disease prevalence, access to health services, among other variables. The average values and variances of the indicators were taken into account, in addition to a 20% non-response rate [11]. The expected sample was 108,525 households, and data were collected from 94,114 households, with a 6.4% non-response rate [11]. A total of 90,846 individuals were interviewed [13]. The NHS is representative of urban and rural areas, large regions of Brazil, federative units, state capitals and metropolitan regions [11].

In this investigation, data from 4,336 adolescents and 6,124 young adults (11.5% of the NHS population) were analyzed.

### Data collection

NHS data collection occurred during household visits. The questionnaire used was validated by specialists and applied by trained professionals, using mobile data collection devices for the interviews [11]. The instrument included questions about sociodemographic characteristics and risk and protective factors for NCDs, among other variables of interest.

### Measurements and variables

#### Dependent variables

In this study, the following indicators were used as dependent variables, categorized dichotomously into “0” No and “1” Yes:

- ***Prevalence (%) of tobacco smokers***: (number of individuals who use tobacco products that emit smoke, regardless of the amount consumed, frequency, and duration/number of individuals interviewed) *100. Users of tobacco products that emit smoke were considered to be individuals who answered positively to the question: “*Do you currently smoke any tobacco products*?” [14, 15].
- ***Prevalence (%) of alcohol use once or more per month***: (number of individuals who consumed at least one alcoholic drink in the past 30 days/number of respondents) *100. It was considered in the numerator the individuals who answered that they consumed alcoholic beverages once or more a month in the question: “*How often do you usually consume alcoholic drinks*?”. A standard drink was defined as the equivalent of a can of beer, a glass of wine, a shot of *cachaça*, whiskey or any other distilled alcoholic beverage [15, 16].
- ***Prevalence (%) of alcohol abuse in the past 30 days***: (number of individuals who reported binge drinking in the past 30 days/number of respondents) *100. Alcohol abuse was defined as an individual who consumed five or more alcoholic drinks on a single occasion, as measured in the following question: “*In the past 30 days*, *have you consumed five or more alcoholic drinks on a single occasion*? [15, 16].
- ***Prevalence (%) of insufficient fruit and vegetable consumption*:** (number of individuals who consume at least 25 servings of fruits (including juice) and vegetables and at least five fruits (including natural juice) and five vegetables per week/number of individuals interviewed) *100. The amount consumed was measured by summing the number of servings of fruits and vegetables per week, which is approximately equivalent to the daily consumption of five servings. The consumption was estimated by the questions: “*How many days a week do you usually eat fruit*?”, “*In general*, *how many times a day do you eat fruit*?”, “*How many days a week do you usually drink natural fruit juice (including frozen fruit pulp)*?”, “*How many days a week do you usually eat at least one type of vegetable (excluding potatoes*, *cassava or yams) such as lettuce*, *tomato*, *cabbage*, *carrots*, *chayote*, *eggplant*, *zucchini*?”; “*In general*, *do you usually eat this type of vegetable*, with options for answers: *Once a day (at lunch or dinner)*, *twice a day (at lunch or dinner) or three times or more per day*” [15].
- ***Prevalence (%) of regular consumption of soft drinks or artificial juices***: (number of individuals who consume soft drinks or artificial juices at least five days a week/number of individuals interviewed) *100. This indicator was measured by the following questions: “*How many days a week do you usually drink juice in a box or can*, *or powdered soft drink*?” and “*How many days a week do you usually drink soda*?” [15].
- ***Prevalence (%) of ultra-processed food consumption***: (number of individuals who consumed five or more ultra-processed food groups on the day before the survey, such as: chocolate milk beverages or flavored yogurt; packaged snacks or salted crackers; sweet or stuffed cookies or packaged cake; sausage, wiener, mortadella or ham; among others/number of individuals interviewed) *100. The questions used to measure this indicator were: “*Yesterday*, *did you drink or eat*: *Soda; Fruit juice in a box or can or powdered soft drink; Chocolate milk beverages or flavored yogurt; Packaged snacks or salted crackers; Sweet or stuffed biscuit/cookie or packaged cake; Ice cream*, *chocolate*, *jelly*, *flan or other industrialized dessert; wiener*, *sausage*, *mortadella or ham; Loaf bread*, *hot dog or hamburger bread; Margarine*, *mayonnaise*, *ketchup or other industrialized sauces; Instant noodles*, *packaged soup*, *frozen lasagna*, *or other ready-to-eat frozen food*”. The response options were “yes” or “no” and UPF consumption was calculated by summing up the positive responses to consumption among the UPF subgroups [15].
- **Prevalence (%) of leisure-time physical inactivity**: (number of individuals who reported practicing less than 150 minutes per week of light or moderate intensity, or 75 minutes per week of vigorous-intensity physical activity/number of individuals interviewed) *100. This indicator considered the answers to the questions: “*In the last 12 months*, *did you practice any type of physical activity or sport*? *(excluding physiotherapy)*”; “*How many days a week do you (did you) practice physical activities or sports*?”; “*In general*, *on the days you do (you did) physical activities or sports*, *how long does (did) this activity last*?” and “*What is the main type of physical activity or sport that you have practiced*?”. Walking, treadmill walking, hydrogymnastics, localized gymnastics/Pilates/stretching or yoga, swimming, martial arts and wrestling, bicycle or stationary bicycle, volleyball, and dance class, among others, were classified as light or moderate intensity practices; or 75 minutes per week of vigorous activities such as running, treadmill running, weight training, aerobics/spinning/step/jump, soccer, basketball and tennis [15, 17].
- ***Prevalence (%) of overweight***: (number of overweight individuals/number of individuals interviewed) *100. Young adult individuals aged 20 to 24 years were classified as overweight when they presented a body mass index (BMI) ≥25 kg/m^2^ was classified as overweight [18], defined as the weight in kilograms divided by the square of the height in meters (kg/m^2^), both self-reported and measured by the questions: “*Do you know your weight*?” with the following options: *Yes*, *which one*? *(in kilograms)* or *I do not know / I do not remember* and “*Do you know your height*?”, with answer options: *If yes*, *which one*? *(in centimeters)* or *I do not know / I do not remember* [15]. For the group of adolescents, the nutritional status of adolescents was assessed using the Z-score as recommended by the World Health Organization [19]. Adolescents with a Z-score ≥1 were classified as overweight [20]. The World Health Organization’s AnthroPlus software was used to calculate the adolescents’ Z-score [19].

Prevalence rates were stratified according to independent demographic and socioeconomic characteristics:

- Sex: male or female.
- Age group (years): stratified into 15–19 years or 20–24 years.
- Race/skin color (self-declared): categorized as white, black, brown, or others, which included the indigenous and yellow categories due to the small number of observations in the sample.
- Education: no education/incomplete middle school, complete middle school/incomplete high school, complete high school/incomplete higher education or complete higher education or more [21].
- Household income (minimum wages): ≤1, 2–3, 4–5 and >5 [21]. In this study, family income was categorized based on the number of minimum wages, with the lowest socioeconomic level, the lowest concentration of income, represented by stratification 1 (one minimum wage), while the highest socioeconomic level, the highest concentration of income, represented by category 5 (five or more minimum wages). A minimum wage was considered as the base salary in Brazil in the reference month of the survey [15]: BRL 998.00 in 2019, BRL 1,039 in January 2020, and BRL 1,045 in February and March 2020 [22–24].
- Living with a partner: no or yes.
- Geographic region: North, Northeast, Southeast, South or Midwest.
- Area of residence: urban or rural.

### Statistical analysis

The NHS adopted a complex sampling design. Thus, sample post-stratification weights were calculated and used for selected households and residents to correct non-response rates and adjusting for the total population of municipalities in Brazil, so that the research sample presents the same age group and sex structure of the Brazilian population. The technique for calculating sample weights was the rake method already described in more detail in previous studies [11].

Statistical analysis involved the description of dependent and independent variables and inferential analysis. Descriptive analysis was performed to describe demographic and socioeconomic characteristics and indicators related to risk factors for NCDs in the total sample and according to sex. The relative frequencies (%) and their respective 95% confidence intervals (95% CI) were used. Pearson’s chi-square test was applied to verify statistical differences in the characteristics of the sample according to the sex of the participant.

Afterwards, multiple Poisson regression was used to examine the associations between each risk factor for NCDs (dependent variables) and demographic and socioeconomic characteristics (independent variables). All Poisson multiple regression models were adjusted for potential confounding factors: sex, age group, race/skin color, education, household income, living with a partner, geographic region, and area of residence. The final models were reported as adjusted prevalence ratio (aPR) assuming a 95% CI. Statistical significance of model variables was established using the Wald chi-square test and *P*-value<0.05 values were considered statistically significant. The collinearity of the independent variables was evaluated using the tetrachoric or polychoric correlation matrix. Collinear variables were those with a correlation coefficient equal to or greater than 0.6 [25]. No variable showed high correlation and, therefore, it was not considered collinear with another. The correlation coefficients ranged from -0.27 (education and area of residence) to 0.43 (age group and living with a partner).

All analyzes were performed using the “survey” package for complex samples of the STATA software (StataCorp LLC, version 16.0, College Station, TX, USA).

#### Ethical aspects

The 2019 NHS was approved by the Research Ethics Committee of the National Health Council, under protocol number 3,529,376/2019. Written consent was obtained from all participants. The microdata are available for access in the research repository [13].

## Results

A total of 10,460 adolescents and young adults were included, 5,001 (47.8%) men and 5,459 (52.2%) women, whose characteristics are depicted in Table 1.

**Table 1:** Description of demographic and socioeconomic characteristics in the sample according to sex. National Health Survey, Brazil, 2019.

Variables	Total (n = 10,460)	Men (n = 5,001)	Women (n = 5,459)	*P*-value*
	% (95% CI)	% (95% CI)	% (95% CI)	
Age group (years)				
15–19	49.5 (47.8–51.2)	49.1 (46.5–51.7)	49.8 (47.5–52.2)	0.683
20–24	50.5 (48.8–52.2)	50.9 (48.3–53.5)	50.2 (47.8–52.5)	
Race/skin color (self-reported)^†^				
White	37.8 (35.9–39.6)	36.5 (34.0–39.1)	38.9 (36.4–41.5)	0.035
Brown	49.1 (47.3–50.9)	50.6 (47.9–53.2)	47.6 (45.2–50.1)	
Black	11.6 (10.6–12.8)	12.1 (10.6–13.7)	11.2 (9.8–2.8)	
Others (yellow or indigenous)	1.5 (1.0–2.4)	0.9 (0.4–1.8)	2.2 (1.3–3.9)	
Education				
No education or incomplete middle school	16.3 (15.0–17.7)	19.8 (17.7–22.0)	12.8 (11.5–14.3)	<0.001
Complete middle school or incomplete high school	36.9 (35.1–38.6)	37.4 (34.9–40.0)	36.3 (33.8–38.8)	
Complete high school or incomplete higher education	42.9 (41.1–44.7)	39.9 (37.5–42.5)	45.9 (43.4–48.5)	
Complete higher education or more	3.9 (3.4–4.6)	2.9 (2.2–3.6)	5.0 (4.1–6.1)	
Household income (minimum wages)^‡^				
≤1	66.1 (64.3–67.8)	64.7 (62.2–67.1)	67.4 (64.9–69.9)	0.355
2–3	27.8 (26.2–29.5)	29.1 (26.8–31.4)	26.6 (24.3–29.0)	
4–5	3.7 (3.1–4.5)	4.0 (3.1–5.1)	3.5 (2.7–4.5)	
>5	2.4 (1.8–3.1)	2.3 (1.6–3.3)	2.5 (1.8–3.5)	
Living with a partner				<0.001
No	79.0 (78.0–80.0)	84.0 (82.0–85.0)	74.0 (72.0–76.0)	
Yes	21.0 (20.0–22.0)	16.0 (15.0–18.0)	26.0 (24.0–28.0)	
Geographic region				
North	10.2 (9.5–10.9)	10.0 (9.1–11.1)	10.3 (9.4–11.4)	0.934
Northeast	29.0 (27.6–30.5)	29.3 (27.3–31.5)	28.7 (26.8–30.8)	
Southeast	39.7 (37.7–41.8)	39.7 (37.0–42.6)	39.7 (36.9–42.4)	
South	13.1 (12.1–14.2)	13.2 (11.7–14.9)	13.1 (11.7–14.6)	
Midwest	8.0 (7.2–8.8)	7.7 (6.7–8.8)	8.2 (7.2–9.4)	
Area of residence				
Rural	16.0 (15.0–17.0)	17.0 (15.0–19.0)	14.0 (13.0–16.0)	0.020
Urban	84.0 (83.0–85.0)	83.0 (81.0–85.0)	86.0 (84.0–87.0)	

95% CI = 95% Confidence Interval;

*Chi-square test adjusted by study design;

^†^Missing for one observation from the men’s group;

^‡^Missing for four observations from the women’s group.

Regardless of sex, the most prevalent risk factors were insufficient fruit and vegetable consumption (92.6%) and leisure-time physical inactivity (43.3%). The prevalence of tobacco smokers, alcohol use once or more per month, and alcohol abuse in the past 30 days were 8.9%, 28.7%, and 18.5%, respectively. The prevalence of regular consumption of soft drinks and/or artificial juices was 17.2%. The prevalence of overweight was 32.5% (Table 2).

**Table 2:** Risk factors for noncommunicable chronic diseases in the sample, according to sex. National Health Survey, Brazil, 2019.

Variables	Total (n = 10,460)	Men (n = 5,001)	Women (n = 5,459)	*P*-value*
	% (95% CI)	% (95% CI)	% (95% CI)	
Tobacco use				
Non-smoker	73.3 (71.7–74.9)	71.7 (69.3–74.0)	75.0 (72.8–77.1)	<0.001
Former smoker	17.8 (16.4–19.3)	15.3 (13.5–17.3)	20.3 (18.3–22.5)	
Smoker	8.9 (7.9–9.9)	13.0 (11.3–14.8)	4.7 (3.8–5.8)	
Alcohol use once or more per month				
No	71.3 (69.6–72.9)	65.7 (63.2–68.2)	77.0 (74.8–79.1)	<0.001
Yes	28.7 (27.1–30.4)	34.3 (31.8–36.8)	23.0 (20.9–25.2)	
Alcohol abuse in the past 30 days				
No	81.5 (80.1–82.8)	75.2 (72.9–77.3)	87.8 (86.3–89.2)	<0.001
Yes	18.5 (17.2–19.9)	24.8 (22.7–27.1)	12.2 (10.8–13.7)	
Recommended consumption of fruits and vegetables				
No	92.6 (91.6–93.5)	94.0 (92.0–95.0)	91.0 (90.0–93.0)	0.009
Yes	7.4 (6.5–8.4)	6.0 (5.0–8.0)	9.0 (7.0–10.0)	
Regular consumption of soft drinks and/or artificial juices				
No	82.8 (81.4–84.2)	82.0 (80.0–84.0)	84.0 (82.0–86.0)	0.074
Yes	17.2 (15.8–18.6)	18.0 (16.0–20.0)	16.0 (14.0–18.0)	
Ultra-processed food consumption				
No	74.0 (73.0–76.0)	73.0 (70.0–75.0)	76.0 (74.0–78.0)	0.029
Yes	26.0 (24.0–27.0)	27.0 (25.0–30.0)	24.0 (22.0–26.0)	
Leisure-time physical inactivity				
No	56.7 (54.9–58.5)	43.0 (41.0–46.0)	70.0 (68.0–73.0)	<0.001
Yes	43.3 (41.5–45.1)	57.0 (54.0–59.0)	30.0 (27.0–32.0)	
Overweight^†^				
No	67.5 (65.8–69.2)	68.2 (65.8–70.5)	66.9 (64.3–69.3)	0.448
Yes	32.5 (30.8–34.2)	31.8 (29.5–34.2)	33.1 (30.7–35.7)	

95% CI = 95% Confidence Interval;

* Chi-square test adjusted by study design;

^†^Missing for 288 observations of women.

Compared to women, men showed a higher prevalence of tobacco smoking (15.3% versus 4.7%; p<0.001), alcohol use once or more per month (34.3% versus 23.0%; *P*-value<0.001), alcohol abuse in the past 30 days (24.8% versus 12.1%; *P*-value <0.001), insufficient fruit and vegetable consumption (94.0% versus 91.0%; *P*-value <0.001) and UPF consumption (27.0% versus 24.0%; *P*-value = 0.029). On the other hand, women had a higher prevalence of leisure-time physical inactivity (70.0% versus 43.0%; *P*-value <0.001) (Table 2).

The regression model showed that the prevalence of tobacco smoking was statistically higher in young adults compared to adolescents and higher in men than women. A negative gradient in the prevalence of tobacco smoking was also observed with decreasing education levels. Prevalence was also higher in the Southeast, South, and Midwest regions in comparison to the North and higher in residents of urban areas compared to rural areas (Table 3).

**Table 3:** Prevalence of tobacco smokers, alcohol use once a month or more, and alcohol abuse in the past 30 days in the sample, according to demographic and socioeconomic characteristics. National Health Survey, Brazil, 2019.

Variables	Tobacco smokers			Alcohol use once or more per month			Alcohol abuse in the past 30 days		
	% (95%CI)	aPR (95%CI)	*P*-value*	% (95%CI)	aPR (95%CI)	*P*-value*	% (95%CI)	aPR (95%CI)	*P*-value*
**Sex**									
Female	4.7 (3.8–5.8)	1.00		23.0 (20.9–25.2)	1.00		12.2 (10.8–13.7)	1.00	
Male	13.0 (11.3–14.8)	2.46 (1.90–3.19)	<0.001	34.3 (31.8–36.8)	1.49 (1.33–1.67)	<0.001	24.8 (22.7–27.1)	2.04 (1.75–2.37)	<0.001
**Age group (years)**									
15–19	6.2 (4.9–7.7)	1.00		20.0 (17.7–22.6)	1.00		12.6 (10.8–14.7)	1.00	
20–24	11.5 (10.2–13.0)	2.56 (1.88–3.51)	<0.001	37.2 (35.0–39.4)	1.76 (1.51–2.05)	<0.001	24.4 (22.6–26.3)	1.86 (1.52–2.28)	<0.001
**Race/color skin (self-reported)**									
White	8.5 (6.9–10.5)	1.00		29.9 (27.3–32.8)	1.00		17.7 (15.6–20.0)	1.00	
Brown	9.0 (7.7–10.6)	1.02 (0.79–1.32)	0.891	26.6 (24.4–29.0)	1.09 (0.96–1.24)	0.179	18.6 (16.7–20.8)	1.14 (0.96–1.34)	0.127
Black	9.8 (7.5–12.8)	1.01 (0.71–1.43)	0.944	33.0 (28.4–37.9)	1.29 (1.08–1.54)	0.006	21.9 (18.4–25.8)	1.27 (1.02–1.57)	0.032
Others (yellow or indigenous)	5.0 (2.4–10.0)	0.84 (0.43–1.65)	0.607	31.0 (12.3–59.0)	1.42 (0.59–3.44)	0.438	11.6 (6.1–20.9)	0.91 (0.52–1.58)	0.741
**Education**									
Complete higher education or more	4.3 (2.1–8.6)	1.00		39.8 (32.3–47.8)	1.00		24.1 (18.5–30.9)	1.00	
Complete high school or incomplete higher education	6.2 (5.1–7.6)	1.61 (0.75–3.45)	0.219	33.1 (30.6–35.7)	1.07 (0.82–1.38)	0.629	20.0 (18.0–22.1)	0.96 (0.72–1.28)	0.787
Complete middle school or incomplete high school	9.0 (7.5–10.8)	3.47 (1.58–7.61)	0.002	23.5 (20.9–26.4)	0.99 (0.77–1.28)	0.961	15.9 (13.9–18.0)	1.00 (0.73–1.37)	0.992
No education or incomplete middle school	16.7 (13.6–20.3)	5.88 (2.74–12.62)	<0.001	26.1 (22.2–30.3)	1.05 (0.85–1.30)	0.638	19.4 (16.0–23.4)	1.10 (0.79–1.53)	0.589
**Household income (minimum wages)**									
>5	4.8 (2.6–8.7)	1.00		51.2 (40.2–62.1)	1.00		22.9 (15.1–33.1)	1.00	
4–5	8.9 (4.5–16.8)	1.45 (0.62–3.38)	0.389	36.8 (28.3–46.2)	0.66 (0.47–0.92)	0.015	25.8 (18.9–34.2)	1.02 (0.61–1.70)	0.937
2–3	8.3 (6.7–10.3)	1.27 (0.66–2.46)	0.468	33.7 (30.5–37.0)	0.61 (0.48–0.78)	<0.001	20.1 (17.6–22.9)	0.80 (0.51–1.23)	0.306
≤1	9.3 (8.1–10.6)	1.40 (0.73–2.71)	0.315	25.3 (23.4–27.3)	0.54 (0.42–0.69)	0.001	17.3 (15.7–19.0)	0.75 (0.48–1.16)	0.190
**Living with a partner**									
No	8.2 (7.2–9.3)	1.00		27.5 (25.6–29.5)	1.00		17.4 (16.0–19.0)	1.00	
Yes	11.4 (9.1–14.1)	1.06 (0.79–1.44)	0.696	33.0 (29.9–36.3)	1.07 (0.94–1.22)	0.324	22.8 (19.9–25.9)	1.18 (0.98–1.41)	0.079
**Geographic region**									
North	6.2 (5.1–7.6)	1.00		18.2 (15.7–21.0)	1.00		15.7 (13.3–18.4)	1.00	
Northeast	6.2 (5.2–7.3)	0.95 (0.73–1.22)	0.675	22.7 (20.6–25.1)	1.23 (1.04–1.47)	0.018	17.9 (16.0–20.0)	1.13 (0.93–1.37)	0.223
Southeast	10.4 (8.5–12.7)	1.87 (1.40–2.49)	<0.001	32.3 (29.1–35.8)	1.64 (1.36–1.97)	<0.001	18.5 (16.0–21.3)	1.10 (0.87–1.38)	0.429
South	11.7 (9.2–14.7)	1.99 (1.45–2.72)	<0.001	39.7 (35.4–44.1)	2.06 (1.70–2.49)	<0.001	21.6 (18.0–25.6)	1.30 (1.01–1.67)	0.038
Midwest	9.9 (7.7–12.7)	1.65 (1.21–2.26)	0.002	27.4 (23.8–31.3)	1.40 (1.15–1.71)	0.001	19.9 (16.8–23.4)	1.16 (0.92–1.46)	0.204
**Area of residence**									
Rural	6.9 (5.6–8.4)	1.00		21.9 (19.3–24.7)	1.00		13.3 (11.1–15.9)	1.00	
Urban	9.3 (8.2–10.5)	1.50 (1.20–1.88)	<0.001	30.0 (28.1–31.9)	1.18 (1.03–1.35)	0.018	19.5 (18.0–21.1)	1.50 (1.23–1.83)	<0.001

95% CI = 95% Confidence Interval; aPR = Adjusted Prevalence Ratio;

*Wald chi-square test.

Regarding alcohol use once or more per month and alcohol abuse, the prevalence was statistically higher in young adults compared to adolescents and in men compared to women. The prevalence of these indicators was also higher in Black participants compared to Whites and in residents of urban areas. There was a reduction in the prevalence of alcohol use once or more per month with decreasing household income. Lastly, the prevalence of alcohol use once or more per month was higher in the Northeast, Southeast, South and Midwest regions than in the North. The South had a higher prevalence of alcohol abuse in comparison to the North (Table 3).

Men have a higher prevalence of insufficient fruit and vegetable consumption when compared to women. The results also revealed an increase in prevalence with decreasing education levels (Table 4).

**Table 4:** Prevalence of insufficient fruit and vegetable consumption, regular consumption of soft drinks or artificial juices, and consumption of ultra-processed foods in the sample, according to demographic and socioeconomic characteristics. National Health Survey, Brazil, 2019.

	Insufficient fruit and vegetable consumption			Regular consumption of soda or artificial juices			Ultra-processed food consumption		
Variables	% (95% CI)	aPR (95%CI)	*P*-value*	% (95% CI)	aPR (95%CI)	*P*-value*	% (95% CI)	aPR (95%CI)	*P*-value*
**Sex**									
Female	91.3 (89.7–92.7)	1.00		16.0 (14.2–17.9)	1.00		24.0 (22.0–26.1)	1.00	
Male	93.9 (92.5–95.0)	1.01 (1.00–1.02)	0.047	18.4 (16.5–20.4)	1.13 (0.97–1.33)	0.120	27.4 (25.2–29.8)	1.14 (1.01–1.30)	0.037
**Age group (years)**									
15–19	92.8 (91.1–94.2)	1.00		16.9 (14.9–19.2)	1.00		27.5 (25.2–29.9)	1.00	
20–24	92.4 (91.2–93.5)	1.00 (0.99–1.01)	0.583	17.4 (15.7–19.3)	1.14 (0.94–1.38)	0.197	24.0 (22.1–25.9)	0.91 (0.79–1.04)	0.171
**Race/skin color (self-reported)**									
White	92.2 (90.2–93.8)	1.00		17.0 (14.8–19.5)	1.00		27.7 (25.0–30.5)	1.00	
Brown	93.1 (91.9–94.2)	1.00 (0.99–1.01)	0.655	15.9 (14.1–17.8)	1.04 (0.86–1.25)	0.719	24.4 (22.6–26.3)	1.01 (0.88–1.16)	0.902
Black	92.6 (89.8–94.7)	1.00 (0.98–1.01)	0.581	22.3 (18.6–26.5)	1.38 (1.10–1.73)	0.005	24.3 (20.3–28.7)	0.98 (0.80–1.21)	0.868
Others (yellow or indigenous)	85.4 (56.5–96.3)	0.96 (0.87–1.07)	0.475	23.9 (9.7–47.9)	1.49 (0.64–3.49)	0.358	29.5 (13.9–52.2)	1.12 (0.57–2.20)	0.742
**Education**									
Complete higher education or more	81.3 (74.2–86.8)	1.00		11.2 (6.8–18.0)	1.00		20.8 (15.0–28.3)	1.00	
Complete high school or incomplete higher education	92.4 (90.9–93.8)	1.05 (1.01–1.09)	0.008	16.1 (14.2–18.2)	1.63 (0.99–2.68)	0.055	25.2 (22.9–27.6)	1.23 (0.89–1.71)	0.211
Complete middle school or incomplete high school	92.6 (90.7–94.2)	1.05 (1.01–1.09)	0.011	19.1 (16.9–21.5)	2.23 (1.32–3.76)	0.003	27.5 (25.0–30.1)	1.34 (0.95–1.89)	0.100
No education or incomplete middle school	95.7 (93.9–97.0)	1.06 (1.02–1.10)	0.001	17.0 (13.9–20.7)	2.18 (1.29–3.71)	0.004	24.2 (21.0–27.7)	1.29 (0.90–1.86)	0.169
**Household income (minimum wages)**									
>5	79.0 (65.3–88.2)	1.00		9.3 (5.4–15.7)	1.00		21.6 (12.5–34.7)	1.00	
4–5	86.5 (79.3–91.5)	1.04 (0.97–1.11)	0.274	26.8 (18.8–36.6)	2.72 (1.43–5.16)	0.002	29.3 (21.1–39.1)	1.33 (0.72–2.42)	0.360
2–3	92.3 (90.2–94.0)	1.07 (1.00–1.14)	0.062	20.1 (17.5–23.0)	1.92 (1.09–3.38)	0.024	29.4 (26.2–32.8)	1.32 (0.78–2.22)	0.304
≤1	93.6 (92.4–94.6)	1.07 (1.00–1.14)	0.050	15.7 (14.2–17.3)	1.62 (0.92–2.84)	0.094	24.1 (22.5–25.9)	1.20 (0.72–2.01)	0.488
**Living with a partner**									
No	92.4 (91.2–93.6)	1.00		16.8 (15.3–18.4)	1.00		26.2 (24.4–28.0)	1.00	
Yes	93.2 (91.8–94.4)	1.00 (0.99–1.00)	0.335	18.5 (15.9–21.5)	1.14 (0.94–1.40)	0.189	24.0 (21.6–26.7)	0.98 (0.86–1.13)	0.809
**Geographic region**									
North	93.6 (91.8–95.0)	1.00		13.4 (11.5–15.7)	1.00		23.6 (21.0–26.3)	1.00	
Northeast	93.3 (91.8–94.5)	1.00 (0.99–1.01)	0.577	11.9 (10.5–13.4)	0.89 (0.73–1.08)	0.240	20.4 (18.5–22.5)	0.88 (0.76–1.02)	0.081
Southeast	91.6 (89.2–93.5)	1.00 (0.98–1.01)	0.508	21.3 (18.5–24.5)	1.44 (1.16–1.78)	0.001	27.5 (24.6–30.7)	1.06 (0.91–1.25)	0.456
South	94.4 (92.5–95.8)	1.01 (0.99–1.02)	0.220	16.9 (14.0–20.3)	1.15 (0.89–1.49)	0.295	35.5 (31.6–39.6)	1.39 (1.17–1.66)	<0.001
Midwest	91.0 (88.3–93.1)	0.99 (0.98–1.01)	0.318	20.8 (17.0–25.2)	1.38 (1.09–1.77)	0.009	22.4 (19.2–26.0)	0.87 (0.72–1.06)	0.171
**Area of residence**									
Rural	94.6 (92.8–96.0)	1.00		8.4 (6.6–10.6)	1.00		15.4 (13.3–17.9)	1.00	
Urban	92.2 (91.0–93.3)	1.00 (0.99–1.01)	0.335	18.8 (17.3–20.5)	1.98 (1.54–2.54)	<0.001	27.6 (25.9–29.4)	1.70 (1.44–2.00)	<0.001

95% CI = 95% Confidence Interval; aPR = Adjusted Prevalence Ratio;

*Wald chi-square test.

As for the regular consumption of soft drinks or artificial juices, a higher prevalence was found in individuals with lower education level and of black race/skin color compared to whites. Notably, the household income followed a pattern like that verified in education, with higher prevalence in individuals with lower family incomes. The prevalence was also higher in residents of urban areas compared to rural areas. Finally, a higher prevalence was recorded in the Southeast and Midwest regions than in the North (Table 4).

With respect to UPF consumption, men, urban residents, and from the South had a higher prevalence of this behavior in comparison to women, rural residents, and from the North, respectively (Table 4).

The prevalence of leisure-time physical inactivity was higher in young adults compared to adolescents and lower in men compared to women. It was also higher in individuals of other races/skin colors (yellow or indigenous) than in whites. A negative gradient in the prevalence of physical inactivity with decreasing education levels and household income was observed. Higher prevalence rates were also reported in individuals living with a partner and residents of the Southeast, South, and Midwest regions. In turn, a lower prevalence was found for urban residents than rural residents (Table 5).

**Table 5:** Prevalence of leisure-time physical inactivity and overweight in the sample, according to demographic and socioeconomic characteristics. National Health Survey, Brazil, 2019.

Variables	Leisure-time physical inactivity			Overweight		
	% (95%CI)	aPR (95%CI)	*P*-value*	% (95%CI)	aPR (95%CI)	*P*-value*
**Sex**						
Female	70.4 (68.2–72.6)	1.00		33.1 (30.7–35.7)	1.00	
Male	43.2 (40.6–45.8)	0.84 (0.82–0.86)	<0.001	31.8 (29.5–34.2)	0.99 (0.89–1.09)	0.798
**Age group (years)**						
15–19	53.8 (51.1–56.6)	1.00		25.1 (22.8–27.6)	1.00	
20–24	59.5 (57.3–61.8)	1.05 (1.03–1.08)	<0.001	39.7 (37.5–41.9)	1.42 (1.26–1.60)	<0.001
**Race/skin color (self-reported)**						
White	55.5 (52.3–58.5)	1.00		32.5 (29.6–35.6)	1.00	
Brown	57.3 (54.9–59.7)	0.99 (0.96–1.01)	0.375	32.3 (30.1–34.5)	1.04 (0.92–1.18)	0.483
Black	55.4 (50.5–60.1)	0.98 (0.95–1.01)	0.184	32.6 (28.2–37.2)	1.02 (0.87–1.21)	0.778
Others (yellow or indigenous)	78.5 (65.1–87.8)	1.09 (1.01–1.18)	0.021	36.5 (16.6–62.4)	1.20 (0.59–2.47)	0.614
**Education**						
Complete higher education or more	39.5 (32.4–47.1)	1.00		42.7 (35.1–50.6)	1.00	
Complete high school or incomplete higher education	55.7 (53.0–58.4)	1.10 (1.04–1.17)	0.001	36.5 (33.9–39.3)	0.93 (0.76–1.14)	0.490
Complete middle school or incomplete high school	55.4 (52.3–58.5)	1.13 (1.06–1.20)	<0.001	27.7 (25.0–30.6)	0.85 (0.68–1.06)	0.145
No education or incomplete middle school	66.5 (62.2–70.4)	1.21 (1.14–1.29)	<0.001	29.8 (26.2–33.7)	0.88(0.69–1.12)	0.293
**Household income (minimum wages)**						
>5	26.5 (18.0–37.1)	1.00		32.3 (23.2–42.9)	1.00	
4–5	36.8 (28.4–46.2)	1.08 (0.98–1.20)	0.128	34.5 (26.4–43.6)	1.05 (0.70–1.57)	0.816
2–3	51.6 (47.7–55.5)	1.18 (1.08–1.28)	<0.001	35.0 (31.6–38.6)	1.05 (0.77–1.42)	0.775
≤1	61.1 (59.1–63.0)	1.23 (1.14–1.34)	<0.001	31.3 (29.3–33.3)	1.02 (0.75–1.39)	0.880
**Living with a partner**						
No	53.2 (51.1–55.2)	1.00		29.8 (27.9–31.9)	1.00	
Yes	70.0 (67.1–72.8)	1.05 (1.03–1.07)	<0.001	42.8 (39.6–46.0)	1.29 (1.16–1.43)	<0.001
**Geographic region**						
North	56.7 (53.6–59.7)	1.00		29.6 (27.0–32.3)	1.00	
Northeast	55.2 (52.7–57.8)	0.99 (0.97–1.02)	0.480	29.0 (26.9–31.3)	0.99 (0.88–1.11)	0.902
Southeast	56.7 (53.0–60.4)	1.04 (1.01–1.07)	0.004	35.1 (31.6–38.7)	1.15 (1.00–1.32)	0.043
South	59.0 (54.7–63.2)	1.05 (1.01–1.08)	0.012	34.2 (30.3–38.2)	1.12 (0.95–1.31)	0.179
Midwest	58.2 (53.6–62.8)	1.04 (1.01–1.08)	0.019	32.7 (28.2–37.5)	1.06 (0.89–1.25)	0.514
**Area of residence**						
Rural	62.7 (59.4–65.8)	1.00		28.0 (25.1–31.1)	1.00	
Urban	55.6 (53.6–57.6)	0.97 (0.95–0.99)	0.004	33.3 (31.4–35.3)	1.10 (0.96–1.25)	0.157

95% CI = 95% Confidence Interval; aPR = Adjusted Prevalence Ratio;

*Wald chi-square test.

Lastly, the estimates demonstrated that the prevalence of overweight was higher in young adults compared to adolescents, among those living with a partner and residents of the Southeast region compared to other Brazilian regions (Table 5).

## Discussion

### Main results

This study revealed a high prevalence of risk factors for NCDs in Brazilian adolescents and young adults. Regardless of sex, the highest frequencies were found for insufficient fruit and vegetable consumption and leisure-time physical inactivity. Men had a higher prevalence of several factors, such as smoking, alcohol use once or more per month, alcohol abuse, insufficient fruit, and vegetable consumption, and UPF consumption. In turn, men had a lower prevalence of physical inactivity. Regression models indicated differences in the associations found for each risk factor. Young adults aged 20–24 years, male, with low education and income levels, black race/skin color, and urban residents were positively associated with most factors. Household income showed an inverse association with alcohol use. Urban area residents also had an inverse association with physical inactivity. Having a partner was associated with three risk factors. Inequalities between Brazilian regions were found for seven of the nine risk factors analyzed. Among them, the Southeast and South regions, despite being more socioeconomically developed, presented the highest magnitudes [24] in unhealthy lifestyles in adolescents and young adults.

### Smoking

Smoking is a well-known risk factor for multiple NCDs, particularly cancers, cardiovascular diseases, and chronic respiratory diseases [1]. Globally, 90% of adult daily smokers first tried smoking before the age of 18, which can lead to the development of NCDs in the future [4]. Our findings showed a prevalence of current tobacco smokers of 8.9%, lower results than those found in the general Brazilian population (12.6%) [14]. In fact, a trend study of indicators related to smoking in Brazil, pointed out a lower prevalence among young people and a higher prevalence in adults [26]. The smaller magnitude of smoking among younger age groups is consistent with educational measures in schools and the total guarantee of cigarette advertising, confident for the drop in the number of young smokers [27]. Likewise, by the greater impact of regulatory measures implemented in Brazil on the population of adolescents and young adults, such as the increase in taxes and prices on tobacco products, prohibiting smoking in public places, inclusion of warnings about the risks of smoking and prohibition of tobacco advertising, sponsorship, and promotion [28]. Our data were also higher than those found in Brazilian students aged 13 to 17 years according to PeNSE in 2019 (6.8%), although methodological differences between both studies must have contributed to these outcomes [8]. PeNSE was conducted with adolescent students and, therefore, does not generalize to non-school individuals [8], unlike the present work. Besides that, PeNSE investigates risk factors in a younger age group (13–17 years) [8], which tends to show a reduced prevalence of smoking compared to older age groups. Finally, it measures the tobacco consumption indicator in the past 30 days prior to the survey [8], while this study uses the prevalence of current tobacco smokers without specifying the period of time.

Men had a higher prevalence of smoking than women, a result similar to that found in previous works in adolescents and young adults [14, 29, 30]. The difference in smoking prevalence between sexes may reflect the influence and interaction between culturally based and gendered norms within different countries and communities [29]. For a long time, women were stigmatized for consuming tobacco products, especially in societies that portray the use of this substance as something inappropriate for women. Smoking is also seen as acceptable and as a symbol of status/social power for men, explaining the higher smoking prevalence in this subgroup [30]. This difference can also be justified by the lower exposure of men to tobacco regulatory measures in Brazil [28]. However, the understanding of the reasons for the differences between the sexes in smoking prevalence should be investigated in further studies, with a qualitative and quantitative approach and including different scenarios. Despite these differences, a prior study conducted in Brazil between 2006 and 2020 has shown a relative reduction in smoking prevalence in men, but stability in women [29], suggesting that increased participation in the labor market, autonomy, and changes in cultural norms and gender roles may contribute in the future to the greater magnitude of smoking in women [29, 30].

Smoking has been shown to be more prevalent in the population with less education [31, 32]. The present investigation found that adolescents and young adults from the lowest education levels had the highest smoking prevalence. Education influences smoking initiation by adolescents and young adults [31]. Less educated individuals are less likely to have access to health education and knowledge about smoking and its health effects, in addition to less use of smoking cessation programs [31, 32]. Furthermore, less educated individuals are less likely to have access to the impacts of smoking reduction interventions, such as regulatory frameworks [31, 32]. Lastly, education can be considered a proxy for socioeconomic status [33], indicating greater purchasing power of individuals with higher education level to acquire tobacco products. These factors may explain the higher smoking prevalence among adolescents and young adults with lower education levels.

This study showed a higher smoking prevalence among urban residents compared to rural residents. Few studies have analyzed the differences in the magnitude of smoking in these two environments, especially the reasons for these inequalities. Previous reports have shown conflicting results. The findings of 2013 and 2019 National Health Survey, for example, described that smoking prevalence in the Brazilian adult population is higher among individuals from rural areas [15, 34]. As found in the general population, surveys carried out in adolescents and young adults out in different countries have observed a higher prevalence of smoking in residents of rural areas compared to urban areas [35, 36]. In addition to the associated factors common to the urban area (for instance: low socioeconomic status, low education level, and exposure to tobacco industry marketing), some works have shown that cultural determinants, including the values of the so-called “rural culture”, which accept and encourage tobacco use [37], has contributed to the increase in the prevalence of this substance in rural environments. This “rural culture” has been considered a social determinant of health [37]. Moreover, tobacco-producing regions in rural areas, especially in the South and Northeast of Brazil, may increase the probability of smoking among residents of rural areas [38]. Ultimately, rural area residents have easy access to illicit cigarettes and have less access to and less impact on information to raise awareness of the effects of tobacco [39]. In turn, the association between smoking and living in an urban area found in this study can be explained by high urbanization, which has been strongly associated with greater commercialization of tobacco products, aggressive marketing by producing companies, and an unfavorable regulatory environment in several urban areas [40]. Additionally, adolescents and young people in urban areas have higher incomes and education, social determinants significantly associated with access to and use of tobacco products [39, 40].

### Alcohol use

Alcohol use is associated with more than 200 diseases and disorders, including numerous types of cancer, cardiovascular diseases, and alcohol-related disorders [41]. We found a prevalence of alcohol abuse of 18.5%, similar to that estimated in the Brazilian adult population (17.1%) [16]. On the other hand, our findings indicate higher prevalence rates than those reported in Brazilian students aged 13 to 17 years (6.9%), although differences in population and methodologies between studies limit the comparability of results [8].

The majority of works have shown a higher prevalence of habitual and excessive alcohol consumption in men compared to women [16, 42], which is in agreement with the outcomes of this investigation. The difference in the prevalence between sexes is conditioned by multiple factors, such as cultural, social, and psychological implications, similar to those found for other substances, such as tobacco [42]. Across several cultures, drinking alcohol is socially acceptable and synonymous with masculinity, status, and power for men. Women are more likely to be judged negatively for alcohol consumption, especially for binge drinking [42]. There is also the hypothesis that men are more exposed to alcohol advertising and marketing strategies to promote alcohol consumption than women [43], thereby contributing to a higher prevalence in this subgroup. Nevertheless, trend studies have reported that alcohol consumption among girls is rising, but not among adolescent males and young adults [42]. A survey conducted with Brazilian adults showed a stable trend in the prevalence of alcohol abuse among men and a significant increase among women from 2006 to 2020 [44]. Another research estimated a higher prevalence of alcohol use in adolescent girls [45], suggesting that this population should also be the focus of strategies to reduce this risk factor. The increase in alcohol use among women may be associated with increased autonomy and higher education levels of this population, labor market participation, and changes in social and cultural norms regarding drinking that increased acceptability of its consumption in various societies, besides other biological factors [42, 44].

Investigations carried out in adults have found racial and ethnic disparities in regards to problem drinking, with significant associations between black race/skin color and alcohol consumption or alcohol use disorders [46, 47]. However, this association is dependent on the interaction with sex and/or found in stratified analysis [46]. The literature shows divergences related to the association between race/skin color and alcohol consumption, especially binge drinking [48]. Data from the Brazilian adult population demonstrate that the probability of alcohol abuse is higher in Black individuals compared to White individuals, even after adjusting for confounding demographic and socioeconomic variables [16, 47]. A previous study that examined the associations of intersections between race/skin color, sex, and excessive alcohol consumption in a sample of Brazilian adults showed that the prevalence of alcohol abuse is greater in Brown and Black women compared to White women, a pattern similar to that found in men, even after adjusting for confounding factors [49]. Multiple hypotheses try to explain the higher prevalence of alcohol use among black people. This is possibly due to mechanisms of racial discrimination, which may increase engagement in addictive behaviors [50]. Another hypothesis is that Black individuals continue to have the lowest education levels, household income, and disparities in access to health care [51], factors that may contribute to a higher prevalence of alcohol consumption, although further studies should investigate the ethnographic, cultural and social reasons for differences in magnitude according to race/skin color.

We found an inverse association between household income and alcohol use once a month or more, and binge drinking. This result is similar to that found in a previous study, which showed that higher parental income and higher education are associated with higher rates of binge drinking among adolescents [52]. Socioeconomic status (SES) directly impacts the prevalence of alcohol use. Existing studies have shown that people with greater SES have a higher prevalence of alcohol consumption and substance use in early adulthood, even though the greatest burden of disease affects socioeconomically disadvantaged groups. Household income is positively associated with the quantity and frequency of alcohol use, owing to greater access to alcohol by people with higher SES [52, 53].

We verified a higher prevalence of alcohol use once a month or more and excessive consumption of alcoholic drinks among urban area residents, which is in accordance with previous reports [16, 54]. The greater alcohol consumption by urban residents can be explained by the high availability, poor or lack of enforcement of local licensing policies on the purchase of alcoholic beverages in cities by adolescents, marketing strategies in cities and frequent alcohol use in urban social environment, providing a stimulus for greater consumption [55]. However, the association between urban environment and alcohol use is controversial and should be the focus of future surveys. Studies conducted in high-income countries showed that alcohol use is more prevalent in rural areas [56, 57]. Differences in enforcement and alcohol availability between countries explain, in part, the divergences found [55]. Cultural values, which include greater acceptability of alcohol use, family interactions that favor consumption, and lower risk perception by rural area residents, may explain the associations between rural environment and alcohol use found in some studies [54, 56].

### Unhealthy eating

This work investigated the association between three indicators of unhealthy eating and multimorbidity. Evidence showed that an increased and regular consumption of fruits and vegetables reduces the risk of several diseases, such as cardiovascular diseases and type 2 diabetes mellitus, and may also prevent overweight and obesity [58]. Recent studies have also found a strong association between UPF consumption, all-cause mortality, and the development of numerous diseases, including hypertension, cardiovascular diseases, type 2 diabetes mellitus, cancer, overweight, and obesity [59]. Excessive consumption of sugar-sweetened beverages, such as soft drinks and artificial juices, has also been associated with an increased risk of multiple NCDs [60].

We observed a high prevalence of insufficient fruit and vegetable consumption, UPF consumption, and regular consumption of soft drinks and/or artificial juice, results higher than those found in the Brazilian adult population (87.0%, 14.3% and 17.2%, respectively) [15]. These findings indicate that the markers of unhealthy eating are more frequent in adolescents and young adults compared to the general population, suggesting the vulnerability of these groups to develop NCDs in the future as a result of unhealthy eating habits. Among food groups, UPF consumption has shown an increasing trend in adolescents and young adults. Thus, ultra-processed foods represent a serious public health concern since they are generally calorically dense, rich in sodium, sugar, saturated and trans fats, and low in fiber and protein [59]. The 2015 PeNSE study reported a prevalence of excessive UPF consumption of 75.0%, defined as eating these foods more than seven times a week [61], showing that adolescents frequently consume soft drinks and other sugar-sweetened beverages, sausages, cookies, and pizzas, among other risk food groups.

This study found a higher prevalence of insufficient fruit and vegetable consumption and UPF consumption in men than women. Previous investigations have shown divergent results regarding these associations. Some indicate no differences or differences in prevalence between sexes for UPF consumption [61, 62] and consumption of unhealthy foods among Brazilian adolescents [63]. Other studies demonstrate a higher prevalence of unhealthy eating behaviors among women [64, 65]. Another research showed that men are less likely to consume fruit and vegetables than women [66]. Although mechanisms to explain sex differences in the frequency of high consumption of ultra-processed foods have not been clearly investigated [67], studies show that women place more emphasis on aesthetic values of their bodies compared to men [68]. Thus, they adopt healthier eating habits more frequently. The influence of the media, easy access to fast foods, and the social context can, in turn, reinforce unhealthy behaviors, such as fast-food consumption, more commonly observed in environments shared by men. In fact, food marketing is more likely to influence the food preferences of boys than girls [69].

We verified an association between a lower education level and insufficient fruit and vegetable consumption, in addition to the regular consumption of soft drinks and/or artificial juices. Indeed, previous reports have shown a lower prevalence of these behaviors in individuals or families with less education [66, 70]. Factors such as lower nutritional awareness and knowledge and the risks involved in unhealthy behaviors may partly explain the higher prevalence of these dietary markers in less educated individuals [71, 72]. Education can also be interpreted as a proxy for household income. In this sense, low-income families have more restricted budgets, prioritizing energy dense foods (for example: soft drinks); therefore, fruit and vegetables may be overlooked [66]. Indeed, the low cost of sugar-sweetened beverages and the high cost of fruits can negatively impact diet quality of individuals with lower education levels [71, 72]. The current study also found an association between low household income and the regular consumption of soft drinks and artificial juices. We also observed a positive association between black race/skin color and regular consumption of soft drinks and artificial juice. Despite the scarcity of works investigating and/or exploring differences in the prevalence of this indicator according to race/skin color, evidence has shown that the probability of sugar-sweetened soda consumption is greater in Black individuals [73, 74]. Race/skin color may interact with family income and education. Black populations have lower education levels and household income compared to Whites; therefore, they have lower nutritional knowledge levels and greater consumption probability due to the low cost of these food groups [75].

This work found a positive association between urban area residents and the regular consumption of soft drinks and/or artificial juice and UPF consumption, which is in agreement with previous findings [61, 76]. Urbanization has led to significant changes in the population’s eating habits. Urban area residents have greater accessibility to unhealthy foods, owing to the greater availability and variety of commercial establishments, products and brands [77]. Furthermore, factors related to income growth, increased formal labor force participation of adolescents and young adults, and technological changes that have encouraged a sedentary lifestyle (e.g., smartphone use) facilitate consumption of unhealthy foods in urban areas [78]. Ultimately, other factors, such as access to modern advertising, may contribute to a greater prevalence of consumption of soft drinks and/or artificial juice and UPF in the urban area [78].

#### Physical inactivity

Physical inactivity it is associated with the occurrence of multiple NCDs (eg, cancers, cardiovascular disease, and diabetes) and all-cause mortality, regardless of other risk factors [79].

We found a prevalence of leisure-time physical inactivity of 43.3%, suggesting that a significant sample of Brazilian adolescents and young adults do not practice the minimum recommendation of 150 minutes of moderate-intensity physical activity per week as defined by the World Health Organization (WHO) [80]. If the prevalence found in this study is maintained in the coming years, Brazil will not be able to reach the global target of a 15% relative reduction in the prevalence of physical inactivity by 2030 established by the WHO [80] and the Ministry of Health for the Brazilian population [9]. The prevalence of physical inactivity in adolescents and young adults was higher than that recorded in the Brazilian adult population in 2019 (30.1%) [17]. These results suggest that more adolescents and young adults engage in physical activity.

Our estimates showed a lower prevalence of physical inactivity in women compared to men, which is in accordance with the findings observed in Brazilian adults [17] and in adolescents and young people [81]. Cultural, social, economic, and behavioral factors may explain sex differences in physical inactivity [17]. Several cultures, for example, encourage boys to engage in physical activity, especially in competitive and team sports, while girls are more encouraged to engage in lower-intensity activities. Therefore, girls have less social support for physical activity [17, 81]. Other aspects, such as perceptions of the physical/social environments and physical activity, may differ between sexes and influence the prevalence of physical inactivity [82]. A prior study revealed that interaction with physical activity-promoting environments, such as recreational parks and schools, is greater in girls than boys [82]. This suggests that actions and policies to promote equity in activity practice between sexes are needed for adolescents and young adults.

This investigation showed higher prevalence rates of physical inactivity with lower education levels and household income. These results are consistent with those reported in the literature [17, 83]. Education is associated with greater knowledge of the benefits of physical activity, higher risk perception of the consequences of physical inactivity, and adoption of healthier lifestyles, including the practice of physical activity [84]. Individuals with higher education are more likely to influence their peers (for example: friends and family) to practice physical activity on a regular and consistent basis; thus, the association in this group may also explain the social influence. Education is a component of the SES and a proxy for family income. Therefore, people with a low education level have lower income, less access to resources and opportunities that facilitate the practice of physical activity [17, 84].

We verified an association between having a partner and physical inactivity These findings corroborate previous research conducted in the general population [85]. Individual roles and demands, especially those related to household chores and financial responsibilities performed by individuals living with a partner, may limit the energy to perform leisure-time physical activity [85]. Also, unfavorable living conditions in modern society may offer greater opportunities for sedentary leisure behaviors, especially for individuals with a partner [85]. Regarding the population of adolescents and young adults, there is a lack of studies for comparison with our findings since few population-based studies carried out in Brazil have examined the influence of marital status, physical inactivity, and sedentary behavior. Thus, further in-depth studies should explore the influence of marital status in these populations.

This work found an inverse association between urban area residents and physical inactivity. These results are consistent with prior studies [86–88]. Urbanization greatly impacts the adoption of unhealthy lifestyles, such as sedentary behavior. In turn, residents of urban areas have greater availability of equipment and public leisure spaces (for example: squares, courts, and bike paths) for practicing physical activity. Also, rural residents have lower income, which may contribute to lower levels of knowledge about the risks of physical inactivity and access to mechanisms for the practice of physical activity. Therefore, to mitigate inequalities in the practice of physical activity and sedentary behavior in urban and rural areas, public policies must be addressed [86–88].

### Overweight

Raised body mass index is considered a major risk factor for the global burden of diseases such as cardiovascular diseases, type 2 diabetes mellitus, and some cancers [89] and shows a growing trend in the world and Brazilian adult population with the nutritional transition [90]. The prevalence found in this study was high, lower than that reported for Brazilian adults living in state capitals in 2019 (55.4%) [91]. In fact, the prevalence of overweight is higher in middle-aged adults compared to adolescents and young adults [91]. However, the findings indicate an increasing obesity epidemic in younger age groups [91], which may lead to an increased burden of NCDs in the future, requiring assertive strategies and public policies.

We observed a higher prevalence of overweight in individuals with a partner, as already reported in the available literature [92, 93]. Studies have shown a higher prevalence of unhealthy food consumption, physical inactivity and sedentary behavior in people living with a partner, which contributes to a higher frequency of overweight in this subgroup [85, 93]. This study also found a higher prevalence of overweight in individuals from the Southeast and South regions, corroborating previous studies both in the general population and in adolescents [94, 95], explaining the regional inequalities due to the high frequency of behaviors of risk in the urban environment [96].

### Limitations and strengths

The present study has limitations. First, the cross-sectional nature of our investigation does not allow to establish causality between the independent variables and the risk factors for the NCDs. Second, behavioral data were self-reported, susceptible to memory and response bias due to social desirability, and may be underestimated. However, to reduce memory bias, we used an instrument with a recall time of less than 30 days for the variables. To minimize response bias, the interviews were carried out in a private location, without other family members or participants. Furthermore, the participants were informed about the anonymization of the data. Third, data such as weight and height were self-reported, leading to a potential information bias in the BMI variable, which may have underestimated the prevalence of overweight. Weights and heights measured by standardized methods are more objective and adequate for future studies, although other investigations have shown the validity of using self-reported anthropometric measurements [44]. Lastly, other risk factors were not evaluated in this study, such as the use of electronic cigarettes, other sedentary behaviors (for example: smartphone use), and other eating habits that could affect this population (for example: consumption of salt, sweets, among others), and should be the focus of further research. The absence of a validated food frequency questionnaire (FFQ) for investigating eating habits is also a limitation that should be addressed. As for the investigation of physical inactivity, it is worth mentioning that we do not collect school data from participants. Adolescents and young adults who are in school have regular physical education classes [97], which can reduce the prevalence of physical inactivity and this fact was not considered in the study.

However, our study has several strengths. The sample is population-based, household and non-school, with national representativeness. The generalization is greater than in studies conducted among schoolchildren. This work included data disaggregated by sex and other important covariates in the analysis of determinants [for example: area of residence (rural or urban)]. Also, we analyzed a set of nine factors, expanding and aggregating data on the epidemiology of risk factors in adolescents and young adults. Remarkably, our findings provide evidence that contributes to the monitoring of NCDs risk factors and identification of subgroups with greater prevalence for each factor. These outcomes favor the implementation and better targeting of interventions and public policies for health promotion, disease prevention, and health surveillance in adolescents and young adults.

## Conclusion

In conclusion, the risk factors for NCDs showed high prevalence among Brazilian adolescents and young adults. Higher frequencies were found for insufficient fruit and vegetable consumption and leisure-time physical inactivity. Young adults, males, and individuals with lower education, of black race/skin color, with lower household income, and urban area residents had a higher prevalence for most risk factors. Differences in determinants were verified for some characteristics. For example, individuals with higher incomes had a higher prevalence of alcohol use once or more per month, and rural residents showed a higher frequency of physical inactivity. Having a partner was associated with two risk factors (physical inactivity and overweight). Inequalities between Brazilian regions were found for seven of the nine factors analyzed. The most socioeconomically developed regions exhibited the highest prevalence of most risk factors.

Alarmingly, the high prevalence of the risk factors indicates a potential increase in the burden of NCDs in the future Brazilian scenario. Preventive interventions should consider the differences between sexes, age groups, urbanization, race/skin color, education, income, and regional inequalities in the prevalence of risk factors for NCDs in adolescents and young adults. Future studies should investigate the determinants of risk factors in this population, controlling for other variables (for example: social influence), including other factors, and performing a stratified analysis (for example: by socioeconomic stratum).
